# Giving or Receiving Something for Sex: A Cross-Sectional Study of Transactional Sex among Ugandan University Students

**DOI:** 10.1371/journal.pone.0112431

**Published:** 2014-11-11

**Authors:** Vikas Choudhry, Per-Olof Östergren, Anne-Emmanuelle Ambresin, Emmanuel Kyagaba, Anette Agardh

**Affiliations:** 1 Division of Social Medicine and Global Health, Department of Clinical Sciences, Faculty of Medicine, Lund University, Malmö, Sweden; 2 Multidisciplinary unit for Adolescent Health, Department of Pediatrics, Lausanne University Hospital- CHUV, Lausanne, Switzerland; 3 Department of Dean of Students, Mbarara University of Science and Technology, Mbarara, Uganda; International AIDS Vaccine Initiative, United States of America

## Abstract

**Objective:**

This study sought to determine the prevalence of transactional sex among university students in Uganda and to assess the possible relationship between transactional sex and sexual coercion, physical violence, mental health, and alcohol use.

**Methods:**

In 2010, 1954 undergraduate students at a Ugandan university responded to a self-administered questionnaire that assessed mental health, substance use, physical violence and sexual behaviors including sexual coercion and transactional sex. The prevalence of transactional sex was assessed and logistic regression analysis was performed to measure the associations between various risk factors and reporting transactional sex.

**Results:**

Approximately 25% of the study sample reported having taken part in transactional sex, with more women reporting having accepted money, gifts or some compensation for sex, while more men reporting having paid, given a gift or otherwise compensated for sex. Sexual coercion in men and women was significantly associated with having accepted money, gifts or some compensation for sex. Men who were victims of physical violence in the last 12 months had higher probability of having accepted money, gifts or some compensation for sex than other men. Women who were victims of sexual coercion reported greater likelihood of having paid, given a gift or otherwise compensated for sex. Respondents who had been victims of physical violence in last 12 months, engaged in heavy episodic drinking and had poor mental health status were more likely to have paid, given a gift or otherwise compensated for sex.

**Conclusions:**

University students in Uganda are at high risk of transactional sex. Young men and women may be equally vulnerable to the risks and consequences of transactional sex and should be included in program initiatives to prevent transactional sex. The role of sexual coercion, physical violence, mental health, and alcohol use should be considered when designing interventions for countering transactional sex.

## Introduction

Transactional sex is defined as an exchange of money, favors or gifts in exchange for sexual relations [1], [2]. The term is used to distinguish the informal or less formal exchanges for sex happening within relationships from the formal, immediate sex for money denoted as ‘commercial sex work’ [3].

Transactional sex is a contributing factor to the HIV pandemic, particularly among young girls in sub-Saharan Africa [2], [4], [5]. It often coexists with other risky sexual behaviors like an early sexual debut, multiple concurrent sexual partnerships, and inconsistent condom use [5]–[8]. There is considerable evidence linking transactional sex to undesirable sexual and reproductive health outcomes including sexually transmitted infections (STIs), unintended pregnancies, unsafe abortions and gender-based violence [8], [9]. Previous studies have indicated that transactional sexual relationships involve a number of power dynamics such as age disparities and unequal access to resources, which may lead to unsafe and coercive sexual practices [2], [7], [10], [11].

A study in four sub-Saharan African countries reported transactional sex to be a common practice among more than two-thirds of young women in Ghana, Malawi, and Uganda [4]. About one-third of young men in Ghana and Uganda reported having received gifts from a recent sex partner in exchange for sexual intercourse [4]. Factors associated with transactional sex include age discrepancies: older men are often involved with young girls and called “sugar daddies” and, less often, older women involved with young men and are referred to as “sugar mummies” [11], [12]. This kind of exchange has also been reported between peers [13].

Transactional sex among women has been linked to basic survival and subsistence needs [3], [4], [11], [14]. However, young women in universities also get involved in transactional sex for higher grades, employment opportunities, luxury consumables that raise their status in peer circles, and sometimes access to social networks [3], [10], [15]–[17]. The transactional nature of sexual behavior sometimes begins at a younger age or during one’s school years, but the prevalence increases rapidly at university where lifestyle costs are higher, there is a lack of parental control, and peer pressure urges a certain affluent lifestyle [15], [16].

University authorities in Uganda have acknowledged the prevalence of transactional sex among university students and its role in HIV transmission among this sub-group of young people [18]. However, it is unclear whether students who provide gifts, money or some form of compensation are different from students who receive gifts/money or some form of compensation in exchange for sexual relations. Previous studies often focus solely on women and their motivations for transactional sex. Given the prevalence of STIs, including HIV, among young people in Ugandan universities, the factors that influence transactional sex should be determined in order to develop effective interventions. It is the aim of this study to assess the prevalence of transactional sex and explore the various risk factors associated with it on a university campus in Uganda.

## Methods

### Study design and setting

The cross-sectional study was conducted at the Mbarara University of Science and Technology (MUST) in southwest Uganda in 2010. Ethical approval was granted by the Institutional Review Committee at MUST. The students were approached in classrooms and in student dormitories at MUST, orally informed about the purpose of the survey and were invited to participate. Participation in the survey was voluntary and anonymity was assured. The students who agreed to participate in the survey were required to sign a consent form on the front page of the questionnaire that also included the explanation and justification of the survey.

The survey took place in lecture halls at MUST, with a member of the research team present. Contact details for the principal investigator and a research assistant were also provided, in case any questions or personal concerns would arise while answering the questions. The consent forms and the questionnaires (without identifying information) were collected separately and placed in different boxes in the front of the rooms to maintain anonymity of the survey.

Of the 2706 undergraduate students invited to participate, 1954 gave their consent and completed the questionnaire (72% response rate). Our study was a follow-up to a survey conducted at MUST in 2005 using the same questionnaire [19], [20]. The self-administered questionnaire was in English, and took around 45 minutes to fill in. There were 132 questions addressing alcohol use, social capital, mental health, and sexual relations (including sexual coercion), in addition to basic socio-demographic characteristics. The questionnaire had been developed in 2005 on the basis of themes that emerged from the focus group discussions held with students at MUST [21] and questions from validated instruments in other studies [22].

### Study measures

*Age* was divided in two categories: 1) ≥24 years and 2) <24 years. The cut-off for age was based on World Health Organization (WHO) definition of youth (15–24 years).

*Area of origin* was recorded as ‘rural,’ ‘urban,’ or ‘peri-urban or small town’, then dichotomized into ‘rural’ and ‘urban/peri-urban or small town’.

*Educational level of head of household* was dichotomized so that ‘did not finish primary school’ and ‘completed primary school’ were coded as ‘≤primary school’ and any education above that as ‘>primary school’.

*Heavy episodic drinking* was based on answers to the question “How often do you drink six glasses or more of alcohol on the same occasion?” The responses ‘daily’, ‘every week’, and ‘every month’ were coded as ‘heavy episodic drinking’ while ‘never’ and ‘abstainers of alcohol’ were coded as ‘no heavy episodic drinking’.

*Mental Health* status was measured using the Hopkins Symptom Checklist (HSCL-25), which assesses symptoms of anxiety (10 items) and depression (15 items) during the preceding week, on a scale from 1 (‘not at all’) to 4 (‘extremely’). In addition, 10 items from the Symptom Checklist-90′s (SCL-90) subscale reflecting psychoticism were included. The SCL-90 is a self-reporting instrument for the assessment of psychiatric symptoms on a scale of 0 to 4. For each item students were asked, “How much has this problem bothered or distressed you during the last week, counting today?” Both the HSCL-25 and the SCL-90 have previously been used in Africa, including a population-based study in the same region of Uganda [23], [24]. A standardized mean score for anxiety, depression, psychoticism, and total mental health symptom scores was obtained for each respondent. This was based on the student’s total score for that measure divided by the number of items answered. Following a similar procedure used in a Ugandan setting [24] variables were dichotomized as ‘high score’ (i.e., poor mental health status) versus ‘low score’ (i.e., satisfactory mental health status) whereby an average score was arrived at, based on a median-split of the distributions of the summary scores for each measure.

*Growing up with parents* was based on the question “Which adults did you live with most of the time or years when you grew up?” The options ‘my mother’, ‘my father’, and ‘others’ was categorized into ‘growing up with single parent & others’ while the option ‘mother and father’ was categorized as ‘growing up with both parents’.

*Social participation* was classified on the basis of participation in 12 different social activities in recent months. Based on the median, the total scores of those who answered ‘yes’ (maximum total score 12) were dichotomized into ‘high’ (above the median) and ‘low’ (below the median).

*Victim of physical violence in the last 12 months* was based on responses of ‘yes’ and ‘no’ to the question “Have you at any time in the last 12 months been a victim of physical violence?”

*Ever experienced sexual coercion* was based on a response of ‘yes’ to any of the following questions: “You have been forced to show your sexual organ”, “Someone has forced you to let them touch your sexual organ”, “Someone forced you to let them suck or lick your sexual organ”, “Someone has forced you to let them show you their own sexual organ”, “You have been forced to watch someone masturbate”, “You have been forced to masturbate someone”, “You have been forced to take part in oral sex or to lick someone’s sexual organ”, “You have been forced to take part in sexual intercourse with the penis in the vagina, or someone has inserted an object into your vagina”, or “You have been forced to pose for a sex photo or sex film”. In the absence of any affirmative answer to the above questions, in addition to an affirmative answer to the question “You have not been forced into any of these”, the individual was classified as ‘unexposed’ to sexual coercion. Using this set of questions as a measurement of sexual coercion has been validated in previous studies in Uganda and Sweden [22], [25].

*Age disparity of the transactional partner* was based on the question “ What was the age of the person from whom you last accepted money or some other form of compensation as payment for sex?” The options “ Older than me but not more than ten years older” and “Older than me by more than ten years” was categorised as ‘ Having age disparate transactional partner’ while the option “About the same age as my own” was categorized as ‘Same age transactional partners’.

*Sex of the person whom you last accepted money or some other form of compensation as payment for sex* was categorized as ‘male’ or ‘female’.

### Outcome variables

*Having ever accepted money/gifts for sexual relations* was based on the question “Have you ever accepted money, a gift or some other form of compensation as payment for sexual relations?” The two negative options being ‘No, it never happened to me’, and ‘No, but I have fantasized about it’ were combined as ‘not having ever accepted’ while the positive option ‘yes’ was categorized as ‘having accepted money, gifts or some other form of compensation for sex’.

*Having paid or given a gift or otherwise compensated for sex* was either categorized as ‘yes’ or ‘no’.

### Statistical analysis

The statistical analysis was done using IBM–SPSS Version 20.0. Confidence intervals (CI) were calculated at the 95% level to estimate statistical significance. Descriptive analyses were stratified by sex. Differences in proportions were calculated using chi-square tests.

Bivariate logistic regressions were performed separately for men and women to calculate the crude odds ratios (OR) for the effect of various risk factors on engaging in selling sex and buying sex in exchange for money/gifts.

A step-wise multivariate logistic regression modeling was performed based on the contributions of various factors in bivariate analysis. Gender was adjusted for in the first step, followed by other study variables. All the factors were included in the final model to facilitate comparisons across reporting paying or accepting money, gifts or other form of compensation in return for sex. Effect modification was tested and interaction terms between being victim of physical violence in the last 12 months and gender, and sexual coercion and gender were included in the final multivariate logistic regression model. The results report sexual coercion and being victim of physical violence in the past 12 months by gender to account for the interactions.

## Results

Experience of sexual coercion was high (29%) in both sexes, although a significantly higher proportion of women (35.4%) reported having experienced some form of sexual coercion at some point of time in their lives. A greater proportion of women (54.5%) than men scored high on mental health scores, indicating poor mental health status. More men than women (23% vs. 18.5%) reported heavy episodic drinking (HED), while being victims of physical violence in last 12 months was similar among men (10.7%) and women (9.8%) (see Table 1).

**Table 1:** Prevalence of socio-demographic factors, alcohol use, mental health, social participation and experience of sexual coercion and physical violence among a sample of Ugandan university students.

Variables	Categories	All (N = 1954)n (%)	Men (n = 1087)n (%)	Women (n = 867)n (%)	chi-square*p-value*
Age	<24 years	1346 (71.7)	708 (67.4)	638 (77.2)	<0.001
	≥24 years	531 (28.3)	343 (32.6)	188 (22.8)	
Area of growing up	Urban	1067 (55.1)	551 (51.2)	516 (60.9)	<0.001
	Rural	869 (44.9)	526 (48.8)	343 (39.9)	
Educational attainmentof parents	>primary school	518 (27.3)	329 (31.1)	189 (22.5)	<0.001
	≤primary school	1382 (72.7)	730 (68.9)	652 (77.5)	
	Missing	(54)	(28)	(26)	
Living arrangementswhile growing up	Both parents	1020 (52.2)	554 (51)	466 (53.7)	.240
	Single parent/others	934 (47.8)	533 (49.0)	401 (46.3)	
Heavy episodicdrinking	No	1547 (79.1)	840 (77.2)	707 (81.5)	<0.001
	Yes	205 (21.9)	151 (22.8)	54 (18.5)	
	Missing	(202)	(96)	(106)	
Mental healthscore	Low (satisfactory)	920 (50.4)	549 (54.5)	371 (45.5)	<0.001
	High (poor)	904 (49.6)	459 (45.5)	445 (54.5)	
	Missing	(130)	(79)	(51)	
Social participation	High	778 (39.8)	454 (41.8)	324 (37.4)	.051
	Low	1176 (60.2)	635 (58.2)	543 (62.6)	
Ever experienceof sexual coercion	No	1143 (71.0)	671 (76.3)	472 (64.6)	<0.001
	Yes	467 (29.0)	208 (23.7)	259 (35.4)	
	Missing	(344)	(208)	(136)	
Victim of physicalviolence in thepast 12 months	No	1687 (89.7)	930 (89.3)	757 (90.2)	.542
	Yes	194 (10.3)	112 (10.7)	82 (9.8)	
Missing		(73)	(45)	(28)	

Table 2 reports the prevalence of transactional sex by gender. The involvement of men and women differed regarding transactional sex. While more women than men reported having accepted money, gifts or some other form of compensation for sex (15.2% vs. 10.1%), a greater proportion of men reported having paid or given a gift or otherwise compensated for sex (22.7% vs. 6.2%). More than one-third of the students failed to answer any of the questions on transactional sex. Missing cases analysis showed a higher proportion of men than women (41.7% vs. 34.3%) who did not answer the questions on transactional sex and sexual coercion.

**Table 2:** Prevalence of transactional sex among sample of Ugandan university students, with chi square differences by gender.

Sexual Behaviorvariable	Categories	All (N = 1954)n (%)	Men (n = 1087)n (%)	Women (n = 867)n (%)	chi-square*p-value*
Exchanging sex byproviding or receiving gifts,money or compensation	No	897 (74.6)	443 (70.0)	454 (79.8)	<0.001
	Yes	305 (25.4)	190 (30.0)	115 (20.2)	
	Missing	(752)	(454)	(298)	
Exchanging sex byproviding gifts, moneyor compensation	No	1115 (85.1)	534 (77.3)	581 (93.9)	<0.001
	Yes	195 (14.9)	157 (22.7)	38.1 (6.1)	
	Missing	(644)	(396)	(248)	
Exchanging sex byreceiving gifts, moneyor compensation	No	1123 (87.5)	597 (89.9)	526 (84.8)	<0.001
	Yes	161 (12.5)	67 (10.1)	94 (15.2)	
	Missing	(670)	(423)	(247)	
Exchanging sex by bothreceiving and providing gifts,money or compensation*		51 (4.2)	34 (5.4)	17 (3.0)	<0.001

*Missing cases not included in analysis (n = 1202).

A majority of men (77%) and women (79%) who reported having accepted money, gifts or some other form of compensation for sex, had done it more than once. Having an age disparate transactional partner was frequent among women (65%) with transactional partners more often being older men, while men mostly had same age transactional partners (65%). Almost all women (92%) who reported having accepted money, gifts or some other form of compensation for sex had a male partner while one-third of all men (n = 67) who reported the same behavior had transactional partners as men (data not shown).

Table 3 presents the bivariate logistic regression analysis between socio-demographic variables and other factors associated with reporting paying and accepting money, gifts or other form of compensation in return for sex by gender. Being a victim of physical violence in the past 12 months, having experienced sexual coercion and poor mental health status were significantly associated with reporting both, having paid or accepted money, gifts or some other form of compensation for sex for men and women. In addition, heavy episodic drinking among men was significantly associated with reporting having paid or given a gift or otherwise compensated for sex.

**Table 3:** Bivariate associations between socio-demographic and other factors with risk of transactional sex among sample of Ugandan university students, by gender.

	Accepting gifts, moneyor compensation for sex(OR_crude_, 95% CI)*			Paying money, givinggift or compensatingfor sex (OR_crude_, 95% CI)*		
Characteristics	All	Men	Women	All	Men	Women
Age≥24 years	1.34(.94–1.92)	1.06(.62–1.82)	**1.88** **(1.15**–**3.04)**	**2.24** **(1.63–3.10)**	**1.98** **(1.36–2.88)**	**2.00** **(1.00–4.02)**
Rural residencewhile growing up	1.38(.99–1.92)	**2.12** **(1.23–3.67)**	1.10(.70–1.72)	1.21(.90–1.65)	1.10(.77–1.50)	1.02(.53–2.00)
Educational attainmentof parents ≤primaryschool	1.14(.80–1.64)	1.32(.80–2.22)	1.13(.67–1.90)	.94(.66–1.33)	.82(.55–1.21)	.63(.26–1.55)
Heavy episodicdrinking	1.37(.98–1.94)	1.61(.95–2.73)	1.40(.85–2.22)	**2.13** **(1.54–2.94)**	**2.02** **(1.40–2.96)**	1.30(.65–2.60)
Poor mentalhealth status	**3.00** **(2.06–4.42)**	**3.60** **(2.00–6.40)**	**2.50** **(1.50–4.10)**	**2.10** **(1.50–2.90)**	**2.30** **(1.60–3.30)**	**3.85** **(1.65–8.90)**
Growing up withsingle parent or others	**1.67** **(1.20–2.35)**	**1.86** **(1.10–3.13)**	**1.60** **(1.01–2.47)**	1.32(.98–1.80)	1.27(.90–1.83)	1.53(.80–3.00)
Low socialparticipation	1.12(.80–1.60)	.98(.60–1.60)	1.20(.75–1.90)	1.20(.90–1.66)	1.31(.93–1.90)	1.58(.78–3.26)
Victim of physicalviolence in thepast 12 months	**8.67** **(5.75–13.10)**	**12.40** **(6.45–23.90)**	**6.20** **(3.65–10.60)**	**2.02** **(1.46–2.80)**	**2.01** **(1.36–2.98)**	**6.38** **(2.75–14.80)**
Ever experience ofsexual coercion	**3.60** **(2.40–5.40)**	**4.55** **(2.55–8.21)**	**3.00** **(1.70–5.30)**	**3.15** **(2.15–4.65)**	**2.85** **(1.74–4.70)**	**5.20** **(2.50–10.85)**

*Crude Odds Ratio [OR_crude_], 95% Confidence Intervals [CI].

Bold font indicates statistical significance at p<.05.

Table 4 reports the multivariate logistic regression analysis between various factors associated with reporting paying and accepting money, gifts or other form of compensation in return for sex. Women had a higher probability of having accepted money, gifts or some other form of compensation for sex after adjustment for all factors in the final model (OR_adjusted_ 8.13, CI 3.57–18.50). Having accepted money, gifts or some other form of compensation for sex was associated with sexual coercion for men (OR_adjusted_ 10.90, CI 5.08–23.42) and women (OR_adjusted_ 5.06 CI 2.72–9.40) and, with poor mental health status among all students (OR_adjusted_ 1.97, CI 1.20–3.22). Men who had been victims of physical violence in the last 12 months had an association with having accepted money, gifts or some other form of compensation for sex, compared to other men, after adjustment for all potential confounders (OR_adjusted_ 2.20, CI 1.08–4.45).

**Table 4:** Multivariate logistic regression analysis* of transactional sex among sample of university students in Uganda.

	Accepting gifts, money or compensation for sex		Paying money, giving gift or compensatingfor sex	
Characteristics	OR_adjusted_	95% CI	OR_adjusted_	95% CI
Female sex	**8.13**	**3.57–18.50**	**.03**	**.01–.09**
Age≥24 years	1.24	.80–1.94	**1.94**	**1.38–2.85**
Rural residence whilegrowing up	1.43	.82–2.05	1.05	.71–1.56
Educational attainmentof parents ≤primary school	.76	.93–2.22	**.55**	**.35–.85**
Heavy episodic drinking	1.50	.97–2.23	**1.67**	**1.14–2.44**
Poor mental health status	**1.84**	**1.16–2.92**	**2.09**	**1.40–3.13**
Growing up with singleparent or others	1.35	.89–2.05	1.26	.87–1.84
Low social participation	.80	.52–1.20	**1.48**	**1.01–2.17**
Victim of physical violenceover the past 12 months#				
Male (Ref- Male not a victimof physical violence)	**2.20**	**1.08–4.45**	**2.25**	**1.26–4.00**
Female (Ref.- Female not avictim of physical violence)	1.20	.59–2.50	**2.58**	**1.09–6.14**
Ever experience of sexualcoercion##				
Male (Ref- Male with no experienceof sexual coercion)	**10.90**	**5.08–23.42**	1.53	.98–2.40
Female (Ref.- Female with noexperience of sexual coercion)	**5.06**	**2.72–9.40**	**3.50**	**1.42–8.60**

*Adjusted Odds ratio [OR_adjusted_], 95% Confidence Intervals (CI). All variables have been adjusted for each other.

# Sex-specific ORs from interaction between victim of physical violence in last 12 months and sex of individual.

## Sex-specific ORs from interaction between ever experience of sexual coercion and sex of individual.

Bold font indicates statistical significance at p<.05.

Being a male and older age (OR_adjusted_ 1.94, CI 1.38–2.85) was significantly associated with experience in having paid or given a gift or otherwise compensated for sex. Also, being a victim of physical violence in the last 12 months for men (OR_adjusted_ 2.25, CI 1.26–4.00) and women (OR_adjusted_ 2.58 CI 1.09–6.14), poor mental health status (OR_adjusted_ 2.09, CI 1.40–3.13), and heavy episodic drinking (OR_adjusted_ 1.67, CI 1.14–2.44) were associated with experience in having paid or given a gift or otherwise compensated for sex. In the fully adjusted model low social participation was significantly associated with experience in having paid or given a gift or otherwise compensated for sex (OR_adjusted_ 1.48, CI 1.01–2.17).

## Discussion

There is a high prevalence of transactional sex among Ugandan university students with gender playing an important role regarding the type of transaction. Accepting money, gifts or some other form of compensation in exchange for sex was more common among women, while men had a higher prevalence of paying or giving a gift or otherwise compensating in exchange for sex. Experience of sexual coercion for both men and women and being a victim of physical violence in men were both associated with having accepted money, gifts or some other form of compensation for sex. Heavy episodic drinking, poor mental health status, and being a victim of physical violence in last 12 months was associated with experience of having paid or given a gift or otherwise compensated for sex. In addition, women with experiences of sexual coercion had a higher likelihood of having paid or given a gift or otherwise compensated for sex in comparison to other women.

Our study found the prevalence of transactional sex to be 25.4%, much higher than shown in national surveys of Uganda [26]. However, it is difficult to make comparison of the prevalence of transactional sex in our study to national estimates because of the different assessment methods, target groups, time periods and the framing of the question on transactional sex [27]. For the purpose of interventions, factors associated with accepting or providing money, gift or some form of compensations for sex may be different, and hence are discussed separately.

### Factors associated with accepting money, gifts or some other form of compensation for sex

Being a woman, having poor mental health status, and experience of sexual coercion were significantly associated with reporting having accepted money, gifts or some other form of compensation for sex. Our results are congruent with findings from other studies in Africa including Uganda, which have found that women who have had coercive experiences were more likely to exchange sex for money and other material gifts [4], [28], [29]. Some research shows that young people, especially women who have previously been sexually coerced, are at risk for self-harm or risky sexual behaviors, including involvement in transactional sex, as a coping mechanism [30].

Studies from high-income countries in Scandinavia have shown that among adolescents, males reported accepting money, gifts or compensations for sex more often, which is contrary to our study findings [31], [32]. Our study also found a stronger association between experience of sexual coercion and reporting accepting money, gifts or some other form of compensation for sex for men than women. Prior research shows that men with experiences of sexual coercion, particularly in childhood, often results in adverse health and psychosocial consequences including an increase in risky sexual behaviors, anxiety about sexual orientation and a crisis of masculine identity [33]. In high-income countries, selling sex by young men often involves older men on the buyers’ side, in contexts of alcohol and drug use, and is associated with psychosocial problems among these young men [31], [32]. Our study cannot determine whether the male behavior of accepting money, gifts or some other form of compensation for sex is a part of sexual exploration, survival need, or an established form of commercial sex work. Nevertheless, our study suggests that males reporting having accepted money, gifts or some other form of compensation for sex, in universities may have a profile, which is particularly vulnerable. Further studies may lead to a better understanding of the profile of young men reporting behaviors of having accepted money, gifts or some other form of compensation for sex and the associated risks.

Our results indicate that men who have been victims of physical violence in the last 12 months were at higher risks of reporting having accepted money, gifts or some other form of compensation for sex compared to other men. A study in the same setting found that when both male and female students were exposed to violence at the university, this exposure was significantly associated with experiences of sexual coercion [25]. Men and women may experience different types of violence, with more sexual violence in the case of girls and women and more physical violence in the case of men. It appears that men in university who have been victims of physical violence or sexual coercion are as likely to report having accepted money, gifts or some other form of compensation for sex, if not more, than women in university who have had similar experiences. However, in our study sample, exposure to physical violence was less frequent for women, hence it is possible that an association between exposure to physical violence and reporting having accepted money, gifts or some other form of compensation for sex among women was difficult to detect. We need to interpret our finding with extreme caution, as our question on physical violence is likely to have captured some degree of violence victimization only. It is possible to have missed a great deal of intimate partner violence, or violent behavior that a participant, particularly females, may have decided to overlook, or did not consider as violence. Moreover, we cannot rule out the possibility that physical violence and sexual coercion is a regular occurrence in transactional sexual relationships, either due to power asymmetries within these relationships or expectations of providers not being fulfilled [34].

Having experienced a coercive event of sexual or physical violence can impact the mental health of a young person of either sex [25]. Hence it is not surprising that poor mental health status was associated with risk of having accepted money, gifts or some other form of compensation for sex within our sample. Our study could not detect an independent association between heavy episodic drinking and transactional sex although prior research has established that transactional sex is common at local bars where men and women drink heavily [35]. Moreover, alcohol is often used as currency in initiating such relationships [36].

It appears that transactional sex may compound a great deal of HIV risk since, from sexual exchanges alone, experiencing physical, mental, or sexual abuse has been linked to a higher risk of HIV infection and poor sexual and reproductive health outcomes [7], [8], [19].

### Factors associated with paying or giving a gift or otherwise compensating for sex

Being a man and of older age was identified as being associated with higher probability of having paid or given a gift or otherwise compensated for sex. A possible reason why men report providing money or gifts in return for sexual relations may include prevailing masculinity norms namely, having a provider role in relationship where the notion of sexual entitlement is a central part of that role [37]. Research has shown that a part of masculinity norms is having multiple female sexual partners, something that can be achieved through transactional sex [36], [37]. The prevalence of transactional sex is much lower in communities and among individuals characterized by higher gender equity values [6], [38]. Program interventions addressing transactional sex that challenge traditional masculinity norms and promote gender equity norms especially among young men, may be valuable [39].

A study done in Uganda found that men and women who had been exposed to threats of violence or subjected to physical violence were approximately twice as likely to experience sexual coercion [25]. The experience of sexual or physical violence, particularly in childhood, may reduce the ability to form emotionally intimate relationships with members of opposite sex, and as a result may encourage preference for sexual coercion and impersonal transactional sex [40]. Hence, it is not surprising that in our study sample, young women having experiences of sexual coercion or physical violence also had a higher likelihood of reporting having paid or given a gift or otherwise compensated for sex. But, we cannot rule out the possibility of relationships, which involve an anticipation of financial rewards or gifts in exchange for sex, turning physically or sexually violent, if the anticipation of financial rewards or gifts is thwarted [6]. Alternatively, it is likely that women who are providing their sexual partners with money are actually a part of the controlling relationship pattern, which involves demands of money and gifts from the sexual partner to initiate or sustain the relationship. In such a scenario, it would seem that extreme power differentials, in form of structural inequalities such as gender inequality, have an imposing role in such relationships.

Young perpetrators or victims of violence have been shown to have high levels of alcohol and drug use, as well as increased likelihood of engaging in other behaviors which increase the risk of HIV transmission, including transactional sex [41]. The use of alcohol and its relationship to commercial sex work has been established, but it is also likely that heavy episodic drinking is associated with less formal modes of paying or compensating for sex among university students as seen in our study [14], [34]. A great many young people, particularly men, frequent bars and use these venues to ply potential sexual partners with drinks, as has been shown in studies in such venues in South Africa [34]–[36].

### Implications

In the past decade, researchers have identified the possible synergistic co-occurrence of various epidemics and risk factors that contribute additively to increased risk of poor health outcomes, a phenomenon referred to as syndemic [42]. The current study extends our understanding of syndemics to illustrate the existence of the possible co-occurrence of factors like experiencing sexual abuse, physical violence, poor mental health, and participation in transactional sex that may act synergistically to compound the HIV risk among young people in universities. The power dynamics in relationships where there is material exchange for sex can mean limited ability to negotiate safe sex and increased likelihood of riskier sexual situations [1]–[3], [11]. Conditional cash transfer programs have become an increasingly popular approach in HIV prevention through incentivizing safe sex or addressing economic inequalities and lack of educational opportunities, particularly among women [43]. However, these cash transfer interventions influence only an aspect of power asymmetry in form of economic inequalities. There is a need of cash transfer interventions to be to be linked to wider gender transformative interventions that address the structural drivers of HIV like gender inequality. Using schools and university as possible spaces for livelihoods and gender transformative intervention may offer a possible approach for reducing the risks of transactional sex in younger people [43].

### Limitations

The cross-sectional design of our study does not allow analysis of causal relationships between events. We acknowledge that while our study sample is highly selective and our findings may not be generally representative of other young people in Uganda, it is representative of Ugandan university population.

The data used in the study were obtained by retrospective self-reporting that could have resulted in recall bias. Although the survey we conducted was anonymous, the sensitive nature of the items, relating to sexual behavior may have affected the responses we received. Selection bias may exist due to the high rate of non-responses (38%). The prevalence of transactional sex and experiences of sexual coercion among students, particularly men, may have been higher than reported in the questionnaire, since these experiences may be considered socially undesirable to report. If this were so, it should bias the differences in the study sample towards the null. Our study did not include experiences of forced anal sex, which may have led to under reporting of sexual coercion, particularly among men having sex with men and in heterosexual relationships in order to prevent pregnancy.

Nonetheless, we believe the inclusion of young men and comparison of risk factors for selling and buying sex is a key strength of our work. Also, our study results could control for a number of potential confounders in assessing the true relationship between various risk factors and transactional sex.

## Conclusions

The high prevalence of transactional sex necessitates a prevention program among university students. We have indicated that males and females who sell or buy sex have different susceptibility profiles. However, both sexes in Ugandan universities are equally vulnerable to the risks associated with transactional sex and therefore both need to be targeted in prevention programs. Policy and programmatic initiatives should also consider the potential role of transactional sex with regard to STIs including HIV risk. Attention must be given to, the possibly intertwined, roles of sexual coercion, physical violence, alcohol use, mental health, and social environmental factors in shaping sexual behaviors of young people. Treatment and counseling programs for youth who are victims of sexual coercion and physical violence are critical to decrease the risks associated with transactional sex, along with research on protective factors against involvement in transactional sex.
